# The complete chloroplast genome sequence of *Habenaria ciliolaris* (Orchidaceae)

**DOI:** 10.1080/23802359.2019.1692727

**Published:** 2019-11-20

**Authors:** Ming-Kun Chen, Juan Chen, Jie Zhou, Shan-Hu Ma, Qing-Dong Zheng, Tai-Xiang Xie, Ye Ai

**Affiliations:** Key Laboratory of National Forestry and Grassland Administration for Orchid Conservation and Utilization, College of Landscape Architecture, Fujian Agriculture and Forestry University, Fuzhou, Fujian, China

**Keywords:** Chloroplast genome, phylogenetic, Illumina sequencing, *Habenaria ciliolaris*

## Abstract

*Habenaria ciliolaris* is a kind of orchid with ornamental value. In this study, we reported the complete chloroplast genome of *H. ciliolaris*. The complete chloroplast genome is 154,544 bp in length, consists of a pair of inverted repeat (IR, 25,455 bp) regions, a large single-copy region (LSC, 84,032 bp) and a small single-copy region (SSC, 19,602 bp). It contains 179 genes, including 133 protein-coding genes, 38 tRNAs, and 8 rRNAs. A maximum-likelihood phylogenetic analysis demonstrated a close relationship between *H. ciliolaris* and *Habenaria radiata.* This work will be valuable for genetic and phylogenetic studies on *H. ciliolaris*.

*Habenaria* is one of the largest genera in the Orchidaceae family, including about 876 species and mainly distributed in tropical and subtropical areas (Govaerts et al. 2013). According to Flora of China, 58 species of *Habenaria* were reported in China (Zhang et al. 2016). The genus *Habenaria* is not only used as important medicinal plant in China but also has a high ornamental value (Tang 2016). *Habenaria ciliolaris* is a species belonging to the *Habenaria* genera. It grows on a hillside or under the forest at an altitude of 140–1800 m (Chen et al. 2009). However, few data are available regarding the *H. ciliolaris* chloroplast genome. Here, we reported the complete chloroplast genome sequence of *H. ciliolaris*. Our data will be valuable for genetic and phylogenetic studies on *H. ciliolaris.*

The plant material of *H. ciliolaris* was collected from Qinglong waterfall scenic area, Yongtai, Fujian province, China (25°46′23.30″N, 118°57′50.50″E). The voucher specimen is kept at the Herbarium of Fujian Agriculture and Forestry University (specimen code YT-MTYFH).

Total genomic DNA was extracted from fresh leaves using the cetyltrimethylammonium bromide (CTAB) protocol (Doyle and Doyle 1987) and chloroplast genome sequences were analyzed using Illumina Hiseq 2000; raw data were filtered with the NGS QC Toolkit (Patel and Jain 2012); the clean reads were compared with the chloroplast sequence of *Habenaria daitata* (GenBank accession no. NC.035834). We used Version 1.3 for SOAPdenovo assembly (Luo et al. 2012). After assembled, the obtained scaffolds and contigs were assembled into the chloroplast genome by Geneious version 2019.1.1 (Li et al. 2019), then Dual Organellar GenoMe Annotator (DOGMA) (Wyman et al. 2004) was used to annotate chloroplast genome. The complete chloroplast genome of *H. ciliolaris* was submitted to GenBank with the accession number MN.495954.

The complete chloroplast genome of *H. ciliolaris* is 154,544 bp in size, which is composed of a large single-copy (LSC), a small single-copy (SSC), and two inverted repeat (IR) regions of 84,032, 19,602, and 25,455 bp, respectively. It contains 133 protein-coding genes, 38 tRNA genes, and 8 rRNA genes. The complete genome GC content was 37.9%.

Phylogenetic analysis was conducted with 11 complete chloroplast genome sequences of Orchidaceae (*Goodyera schlechtendaliana*, *Goodyera procera*, *Goodyera fumata*, *Anoectochilus emeiensis*, *Goodyera velutina*, *H. ciliolaris*, *Habenaria radiata*, *Habenaria pantlingiana*, *Platanthera japonica*, *Cypripedium formosanum*, and *Paphiopedilum dianthum*), using a maximum-likelihood analysis of MEGA 6.0 (Tamura et al. 2013) with 1000 bootstrap replicates. The phylogenetic tree indicated that *H. ciliolaris* was most closely related to *H. radiata*, as expected (Figure 1).

**Figure 1.**
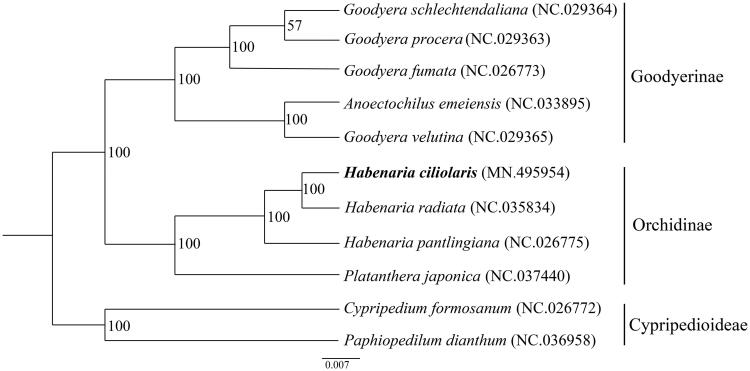
Maximum-likelihood phylogenetic tree of *H. ciliolaris* with 10 complete chloroplast genome sequences of Orchidaceae. Numbers in the nodes are the bootstrap values from 1000 replicates and the position of *H. ciliolaris* is shown in bold. All the sequences were downloaded from NCBI GenBank.
